# Relationship between bioelectrical impedance–derived muscle quality and physical activity, sedentary behavior, or sleep using compositional data analysis (CoDA)

**DOI:** 10.1038/s41598-025-27953-3

**Published:** 2025-12-18

**Authors:** Yujiro Asano, Motohiko Miyachi, Hinako Nanri, Haruka Murakami, Yuko Gando, Takashi Nakagata, Tsukasa Yoshida, Tomohiro Okura, Yosuke Yamada

**Affiliations:** 1 Doctoral Program in Physical Education, Health and Sport Science, Degree Programs in Comprehensive Human Sciences, Graduate School of Comprehensive Human Sciences, University of Tsukuba, 1-1-1 Tennodai, Tsukuba, 305- 8574 Ibaraki Japan; 2 Center for Physical Activity Research, Health and Nutrition, National Institutes of Biomedical Innovation, KENTO Innovation Park NK Building, 3–17, Senriokashinmachi, Settsu, 566-0002 Osaka Japan; 3 Department of Frailty Research, Center for Gerontology and Social Science, National Center for Geriatrics and Gerontology, Obu, 474-8511 Aichi Japan; 4 Faculty of Sport Sciences, Waseda University, 2-579-15 Mikajima, Saitama, 359- 1192 Japan; 5 Laboratory of Gut Microbiome for Health, Microbial Research Center for Health and Medicine, National Institutes of Biomedical Innovation, Health and Nutirition, 7-6-8 Saito-Asagi, Ibaraki, 567-0085 Osaka Japan; 6 Laboratory of Behavioral Physiology, Center for Clinical Nutrition, National Institutes of Biomedical Innovation, Health and Nutirition, KENTO Innovation Park NK Building, 3–17, Senriokashinmachi, Settsu, 566-0002 Osaka Japan; 7 Faculty of Sport and Health Science, Ritsumeikan University, 1-1-1 Nojihigashi, Kusatsu, 525-8577 Shiga Japan; 8 Faculty of Sports Science, Surugadai University, 698 Azu, Hanno, 357- 8555 Saitama Japan; 9 Institute for Active Health, Kyoto University of Advanced Science, Kameoka, Kyoto Japan; 10 Institute of Health and Sport Sciences, University of Tsukuba, 1-1-1 Tennodai, Tsukuba, 305-8574 Ibaraki Japan; 11 R&D Center for Tailor-Made QOL, University of Tsukuba, 1-2 Kasuga, Tsukuba, 305-8550 Ibaraki Japan; 12 Department of Sports and Health Sciences, Graduate School of Biomedical Engineering, Tohoku University, 1-2 Seiryo-cho, Aoba-ku, Sendai, Miyagi Japan; 13 Department of Medicine and Science in Sports and Exercise, Tohoku University, 1- 2 Seiryo-cho, Aoba-ku, Sendai, Miyagi Japan

**Keywords:** Phase angle, Extracellular water, Intracellular water, Sedentary behavior, Moderate-to-vigorous-intensity physical activity, Light-intensity physical activity, Sleep, Bioelectrical impedance spectroscopy., Health care, Medical research

## Abstract

The association between exercise behaviors—including physical activity (PA), light-intensity PA (LPA), moderate-to-vigorous-intensity PA (MVPA), sedentary behavior (SB), and sleep—and muscle quality remains unclear. This study aimed to examine the association between 24-h movement behaviors and muscle quality, as assessed by bioelectrical impedance spectroscopy (BIS). A total of 294 participants were enrolled in the cross-sectional study. LPA, MVPA, and SB were objectively measured using a tri-axial accelerometer. Sleep duration was assessed through a validated self-reported questionnaire. Muscle quality, based on electrical properties, was evaluated using a BIS device including parameters such as phase angle, intracellular-to-extracellular water ratio, membrane capacitance, characteristic frequency. Associations between 24-h movement behaviors and muscle quality derived from electrical properties were examined using compositional multiple linear regression. Additionally, hypothetical time reallocations among different movement behaviors in relation to muscle quality were evaluated using compositional isotemporal substitution. The same analyses were also conducted among participants classified as non-regular exercisers. Longer durations of MVPA were significantly associated with better indicators of muscle quality. Theoretical time reallocation analyses suggested that 30 min from SB, LPA, or sleep to MVPA was associated with approximately 0.5–3.8% higher values of BIS-derived muscle quality indices. This association remained consistent among non-regular exercisers. These cross-sectional findings indicate that greater daily MVPA is associated with a more favorable BIS-derived muscle-quality profile. MVPA is a key component of daily movement that contributes to the maintenance and potential improvement of muscle quality, as evaluated through electrical properties measured using BIS in both regular and non-regular exercisers.

**Supplementary Information:**

The online version contains supplementary material available at 10.1038/s41598-025-27953-3.

## Introduction

Muscle strength and power decline more rapidly with age than skeletal muscle mass^1,2^. Several studies have examined “muscle quality,” which is associated with multiple health outcomes and plays a crucial role in promoting healthy aging^3–5^.

Magnetic resonance imaging (MRI) and computed tomography (CT) are widely used in clinical settings to noninvasively evaluate both muscle quantity and quality. However, high equipment costs, limited portability, and the requirement for highly trained personnel constrain their applicability in community-based environments and large-scale screening programs. In contrast, bioelectrical impedance spectroscopy (BIS) is a noninvasive, portable, and accessible method that yields multiple indicators of muscle quality. BIS offers the advantage of evaluating a range of muscle characteristics, including body cell mass/fat free mass (BCM/FFM), intercellular spacing, cell membrane integrity, extracellular and intracellular water (ICW) distribution, and phase angle (PhA), based on electrical properties not captured by MRI or CT^5^. These indicators are commonly employed as representative markers of muscle quality. Compared to MRI or CT, BIS does not require specialized personnel or costly infrastructure and facilitates rapid, portable, and noninvasive measurements in both community and clinical settings. Additionally, BIS offers unique physiological insights by capturing membrane integrity and fluid distribution through electrical impedance, which anatomical imaging techniques are unable to directly assess. Furthermore, a modified BIS protocol has been developed to facilitate efficient and rapid measurements^6^. Therefore, BIS is anticipated to be widely adopted as a standard assessment tool across diverse clinical and research fields.

Muscle quality, as assessed through electrical properties, has been associated with multiple health outcomes, including muscle strength, physical function, maximal oxygen uptake, and mortality^5,7–10^. Identifying modifiable factors is essential for the maintenance and enhancement of muscle quality. Several studies have demonstrated the beneficial effects of resistance training on muscle quality derived from electrical properties^11–13^. Although resistance training is an effective intervention, its implementation is often hindered by issues such as equipment accessibility, the requirement for trained personnel, and challenges with adherence. A more practical and modifiable factor is the 24-h movement behavior framework, which includes physical activity (PA), sedentary behavior (SB), and sleep. This framework has been recognized as a straightforward yet effective approach to enhancing a range of health outcomes^12,14–16^. Several studies have explored the association between PhA (a marker of muscle quality derived from electrical properties) and SB, PA, and sleep^12,17–19^. However, limited research has investigated the association between general muscle quality derived from electrical properties and movement behaviors, including SB, PA, and sleep.

The co-dependent structure of daily time allocation among movement behaviors must also be considered. As a day comprises only 24 h, increasing time spent on one behavior (e.g., PA) inherently reduces time allocated to another (e.g., SB or sleep). Compositional data analysis (CoDA) is a statistical approach that addresses this co-dependence and is recommended for examining the associations between time-use behaviors and health outcomes^20–22^. Previous studies investigating the association between PA and PhA have not incorporated this co-dependence, potentially leading to inaccurate conclusions^23^.

Therefore, this study examined the associations between SB, intensity-specific daily PA, and overall muscle quality derived from electrical properties, using CoDA to appropriately address their co-dependent nature. Additionally, a subgroup analysis was performed among a sedentary individual who did not engage in regular PA. The hypothesis posited that moderate-to-vigorous-intensity PA (MVPA) would be positively associated with general muscle quality derived from electrical properties, and that relocating time from SB to MVPA would be beneficial.

## Methods

### Participants

This cross-sectional study was conducted at the National Institute of Health and Nutrition in Tokyo. Details of participant recruitment have been reported elsewhere^24^. The inclusion criteria were as follows: (1) measurement of anthropometric variables, (2) assessment of physical activity using accelerometer-based activity monitors, and (3) evaluation of body composition using BIS. Participants with cardiovascular, respiratory, neurological, metabolic, or orthopedic diseases were excluded. The study was approved by the Institutional Review Board of the National Institute of Biomedical Innovation, Health and Nutrition (Approval No. KENEI-102). Written informed consent was obtained from all participants prior to data collection. All study procedures were conducted in accordance with institutional guidelines and relevant ethical regulations.

### Anthropometry and body composition

Body composition was assessed using BIS (SFB7, ImpediMed, Pinkenba, Australia). The fundamental principles and measurement procedures of BIS have been described in detail in previous studies^10,25,26^. Calibration was conducted prior to measurement using precision resistors supplied by the manufacturer, following the manufacturer’s recommendations. Throughout data collection, trained operators continuously monitored the device to prevent measurement errors. Bioelectrical impedance measurements employed a logarithmic distribution of 256 frequencies ranging from 4 to 1,000 kHz using disposable tab-type monitoring electrodes (3 M Red Dot). Participants rested in a supine position for 5 min prior to BIS measurement to facilitate fluid equilibration. The between-day coefficients of variation (CV) for repeated extracellular water (ECW) and intracellular water (ICW) measurements in the laboratory were 2.0% and 3.4%, respectively, while the corresponding intraclass correlation coefficients (ICC^1,3^) were 0.969 and 0.896. All measurements were conducted over a 15-minute period with participants in a supine position, limbs slightly abducted, and palms facing upward. All assessments were carried out by trained personnel using standardized protocols to ensure accuracy and reproducibility. Further details regarding the measurement procedures can be found in previous publications^10,25,26^.

The BIS variables were obtained by measuring the impedance on the right side of the body. The injection electrodes were positioned on the dorsal side of the right foot near the second and third metatarsal joints, and on the dorsal side of the right hand adjacent to the corresponding metacarpal joints. The sensing electrodes were placed at the joint gaps of the radial and ulnar styloid processes and on the right medial and lateral condyles. The analysis parameters included a minimum frequency (R0) of 5 kHz and a maximum frequency (R∞) of 250 kHz. ICW resistance, derived from R0 and R∞ (Ri[0-∞]), was calculated using the formula: 1/([1/R∞] – [1/R0]). ICW resistance from Z5 and Z250 (Zi[5–250]) was calculated as: 1/([1/Z250] – [1/Z5])^27^. R0 and Z5 represented ECW resistance, whereas Ri(0-∞) and Zi(5–250) indicated ICW resistance values. ECW and ICW volumes were estimated using previously established methods^28–30^. Two resistance ratios of ICW to ECW were calculated using Re(0-∞)/Ri(0-∞) and Ze(5–250)/Zi(5–250) values. These ratios were expressed as Re/Ri(0-∞), Ze/Zi(5–250), and ECW/ICW, respectively. Body cell mass (BCM) and fat-free mass (FFM) were obtained using BIS software (Bio-imp, version 5.5.0.1; ImpediMed), and BCM/FFM ratio was calculated. Additionally, membrane capacitance (Cm), characteristic frequency (fc), and PhA were determined using the Cole–Cole model^10,31^. According to previous studies and physiological interpretations, higher values of PhA, Re/Ri, Ze/Zi, Cm, and BCM/FFM were indicative of better muscle quality. In contrast, lower values of Fc and ECW/ICW were associated with more favorable muscle cell function and water distribution. Height and weight were measured, and the body mass index (BMI) was calculated as weight divided by height in meters squared (kg/m²).

### Movement behavior

Average sleep duration was evaluated based on responses to the questions: “In the past month, what time did you usually go to bed?” and “In the past month, what time did you usually get up?” These questions were originally developed based on items from several validated sleep questionnaires and administered separately for weekdays and weekends to more accurately capture habitual sleep duration. Sleep duration was expressed as a proportion of 24 h, and theoretical waking duration was calculated by subtracting sleep duration from 24 h^32^.

PA was measured using a previously validated triaxial accelerometer (Actimarker EW4800; Panasonic, Osaka, Japan). The theoretical framework and algorithm for processing accelerometer data were developed and validated in previous studies and are described elsewhere^33,34^. The device sampled accelerometer output at 20 Hz and applied a low-pass filter to remove signal noise. A three-dimensional vector norm was computed every minute, and acceleration data were stored in 1-min epochs. Participants were instructed to wear the device continuously during waking hours for one month and to remove it during sleep, showering, bathing, or any water-based activity. Participants were included in the analysis if they had a minimum 14 valid days of accelerometer data, regardless of whether those days were consecutive. A valid day was defined as one with more than 10 h of recorded wear time, based on the compositional data analysis framework established in the study^22^. Wear time was verified using accelerometer data in conjunction with a daily self-reported questionnaire. All participants included in the analysis had a minimum 14 valid days, and no cases were excluded due to insufficient wear time or device malfunction.

The metabolic equivalent of tasks (METs) was recorded at 1-minute intervals. MVPA was defined as MET ≥ 3.0, LPA as 1.5–2.9 METs, and SB as any waking behavior characterized by ≤ 1.5 METs, calculated as the difference between total sleep duration and time above 1.1 METs^24,35^. The accelerometer’s objective wearing time excluded acceleration data below 1.1 METs, corresponding to complete stillness (no-signal time). Thus, the device-recorded wearing time could not distinguish between no-signal and non-wearing periods. Accordingly, objective wearing time was calculated as 24 h minus the self-reported sleep duration from the questionnaire. SB was determined by subtracting MVPA and LPA durations from the total wearing time.

### Covariate measurements

The covariates included age, sex, smoking status, BMI, and histories of chronic diseases (diabetes, dyslipidemia, hypertension, and cancer). Age, sex, smoking status, and cancer history were assessed using self-reported questionnaires. Histories of other chronic diseases were determined through self-reported questionnaires, and fasting blood samples were collected from participants after a minimum of 10 h of fasting. A history of dyslipidemia was defined as triglyceride levels ≥ 150 mg/dL^− 1^ and/or high-density lipoprotein cholesterol levels < 40 mg/dL, or ongoing treatment for dyslipidemia. A history of diabetes was defined as a fasting glucose level ≥ 110 mg/dL or current treatment for diabetes. A history of hypertension was defined as systolic blood pressure ≥ 130 mmHg and/or diastolic blood pressure ≥ 85 mmHg, or current treatment for hypertension. Furthermore, participants were classified into regular and non-regular exercise groups based on their responses to the question: “In your daily life, do you walk or engage in equivalent physical activity for at least 1 h per day?”

### Statistical analyses

Statistical analyses were conducted using R version 4.3.2 (R Foundation for Statistical Computing, Vienna, Austria). Statistical significance was defined as *p* < 0.05. Prior to analysis, all raw data were examined to confirm the absence of data entry errors or apparent measurement inaccuracies. Analyses were conducted using the R package compositions (version 2.0.4), in alignment with previous studies employing CoDA to investigate movement behaviors^22^. As the time-use data contained no zero values, zero-replacement procedures were not deemed necessary. All compositional data analyses, including the application of pivot coordinate transformations, were conducted in accordance with this framework. Before analysis, time-use compositions (sleep, SB, LPA, and MVPA) were transformed into isometric log-ratio (ilr) coordinates through pivot coordinate representation. Three ilr coordinates were generated to represent the relative contributions of the various movement behaviors. The first ilr coordinate represented the proportion of time allocated to sleep relative to other movement behaviors. This process was iteratively applied to generate four ilr coordinate systems, each with the first coordinate representing the relative importance of a distinct behavior.

Compositional multiple linear regression analyses were performed to evaluate the relationship between movement behaviors and muscle function derived from electrical properties. The regression model included muscle quality derived from electrical properties as the dependent variable and movement behaviors (expressed as ilrs) as independent variables. Covariates in the model included age (continuous), sex (male/female), BMI (continuous), smoking status (never/quit smoking/current smoking), and medical history of diabetes, dyslipidemia, hypertension, and cancer (all binary: yes/no).

The results of compositional multiple linear regression (i.e., beta coefficients) cannot be interpreted in isolation. Unlike traditional regression analysis, where the difference in the objective variable’s value for a one-unit (min/day) increase in behavior can be directly interpreted, compositional multiple linear regression results do not allow such interpretation. Therefore, to obtain meaningful insights, an additional analysis was conducted. Upon identifying significant relationships between time allocated to each movement behavior, compositional isotemporal substitution^23,32^ was applied in the final model. Compositional isotemporal substitution analysis was performed to examine the association between variations in time spent on movement behaviors and muscle quality derived from electrical properties. This method estimates the theoretical difference in muscle quality based on electrical properties, resulting from increases or decreases in time spent on specific behaviors. Additional methodological details are available in the literature^23^. In this study, two modeling scenarios were employed to estimate the theoretical differences in muscle quality derived from electrical properties. In the first scenario, time allocated to the remaining behaviors (e.g., SB, LPA, and sleep) was proportionally reduced, while time allocated to a target behavior (e.g., MVPA) was increased (i.e., one-to-remaining reallocation). In the second scenario, a fixed duration of time allocated to one behavior (e.g., SB) was reallocated to another behavior (e.g., MVPA), while time allocated to the remaining behaviors, such as LPA and sleep, remained constant. Additional analyses were conducted separately for the non-exercise group. As part of the sensitivity analysis, multiple linear regression was conducted using both unadjusted and adjusted models.

## Results

### Characteristics of the participants

A total of 332 individuals met the eligibility criteria; however, 39 were excluded due to non-participation in accelerometer use (*n* = 34) or incomplete questionnaire and blood sampling data (*n* = 4). The final analysis included 294 individuals (60 men and 234 women; mean age ± standard deviation, 64.7 ± 11.3 years) (Table 1).

**Table 1:** Characteristics of the study participants.

	Regular Ex (*n* = 188)		Non-regular Ex (*n* = 106)		Total (*n* = 294)	
Sex, n (%)						
Women	153	(81.4)	81	(76.4)	234	(79.6)
Men	35	(18.6)	25	(23.6)	60	(20.4)
Age, years (SD)	64.8	(10.6)	64.4	(12.4)	64.7	(11.3)
BMI, kg/m^2^ (SD)	22.5	(2.9)	23	(3.5)	22.7	(3.1)
Smoking status, n (%)						
current smoking	5	(2.7)	5	(4.7)	10	(3.4)
quit smoking	43	(22.9)	22	(20.8)	65	(22.1)
never	140	(74.5)	79	(74.5)	219	(74.5)
Cancer, n (%)						
No	180	(95.7)	91	(85.8)	271	(92.2)
Yes	8	(4.3)	15	(14.2)	23	(7.8)
High blood pressure, n (%)						
No	103	(54.8)	57	(53.8)	160	(54.4)
Yes	85	(45.2)	49	(46.2)	134	(45.6)
Dyslipidemia, n (%)						
No	125	(66.5)	77	(72.6)	202	(68.7)
Yes	63	(33.5)	29	(27.4)	92	(31.3)
Diabetes, n (%)						
No	178	(94.7)	93	(87.7)	271	(92.2)
Yes	10	(5.3)	13	(12.3)	23	(7.8)
Mean time-use composition, min/day (SD)						
Sleep	411	(62.1)	418	(67.1)	414	(63.9)
MVPA	64.8	(37.1)	47	(23.8)	58.4	(34.0)
LPA	364	(93.9)	337	(91.3)	354.0	(93.8)
SB	600	(105.7)	638	(102.7)	614.0	(106.0)
Phase angle, ° (SD)	5.4	(0.8)	5.2	(0.8)	5.3	(0.8)
ECW/ICW, ratio (SD)	0.78	(0.06)	0.80	(0.07)	0.78	(0.06)
Re/Ri (0-∞), ratio (SD)	0.37	(0.06)	0.36	(0.06)	0.37	(0.06)
Ze/Zi (5–250), ratio (SD)	0.24	(0.04)	0.23	(0.04)	0.24	(0.04)
fc, kHz (SD)	51.7	(8.0)	52.7	(9.1)	52.0	(8.4)
Cm, nF (SD)	1.4	(0.5)	1.3	(0.4)	1.4	(0.5)
BCM/FFM, ratio (SD)	0.58	(0.02)	0.57	(0.02)	0.58	(0.02)

Note. Ex: exercise group, SD: standard deviation, BMI: body mass index, MVPA: moderate-to-vigorous-intensity physical activity, LPA: light-intensity physical activity, SB: sedentary behavior, ECW/ICW: estimated extracellular water to intracellular water ratio, Re/Ri (0-∞): extracellular to intracellular water resistance ratio calculated at the estimated frequency of 0 and ∞, Ze/Zi (5–250): extracellular to intracellular water resistance ratio calculated at the frequency of 5 and 250, Fc: characteristic frequency, Cm: membrane capacitance, BCM: body cell mass, FFM: fat-free mass.

### Association between 24-h movement behaviors and electrical property-derived muscle quality in all participants

**Fig. 1:**
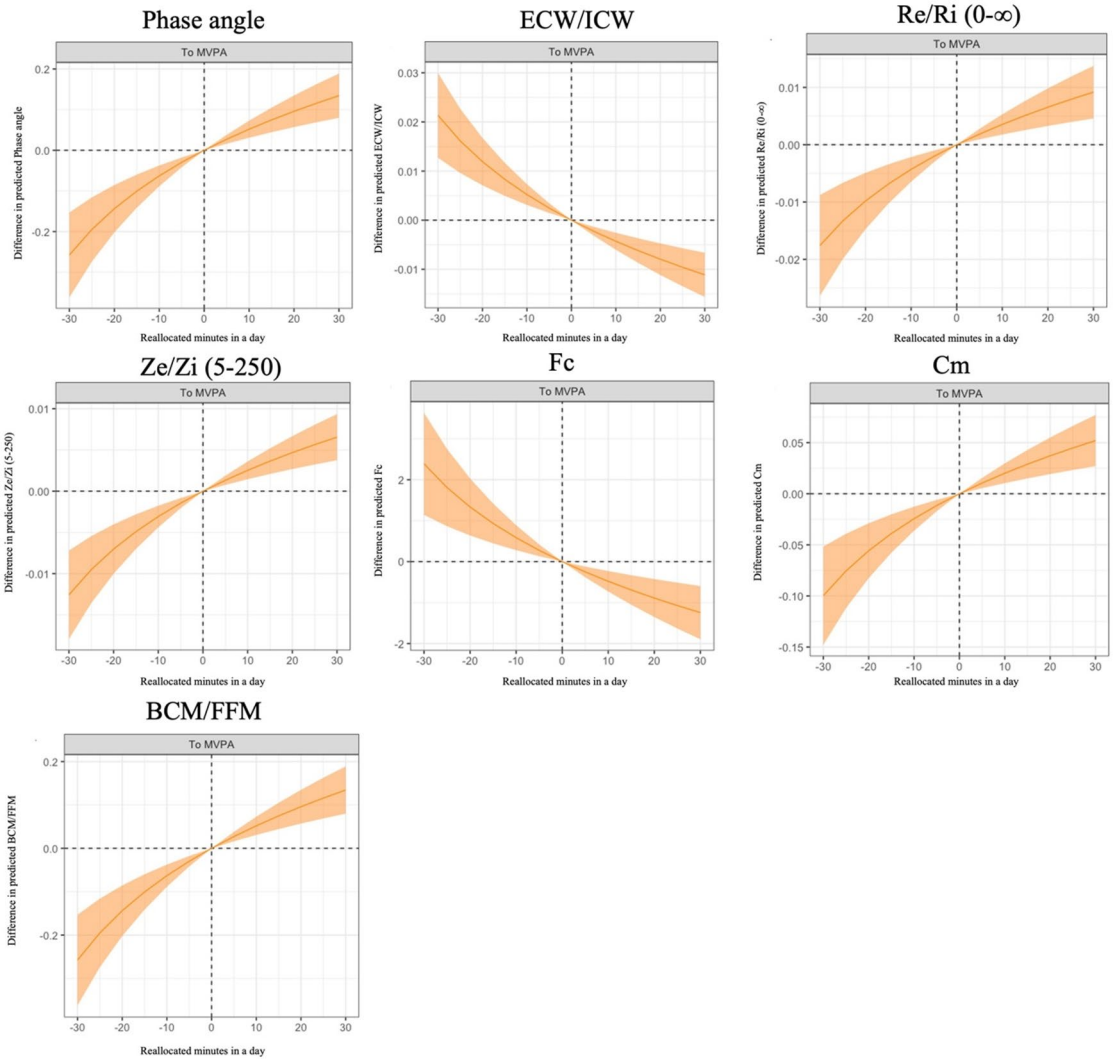
Predicted differences in muscle quality, derived from electrical properties, were assessed by reallocating fixed durations of time between moderate-to-vigorous-intensity physical activity and other behaviors, while maintaining the remaining components at their compositional means. Solid lines represent point estimates, and shaded areas indicate 95% confidence intervals. The analysis based on a regression model that included the following variables as covariates: age, sex, body mass index, smoking status, history of cancer, renal insufficiency, high blood pressure, dyslipidemia, and diabetes. MVPA: moderate- to-vigorous-intensity physical activity, LPA: light-intensity physical activity, SB: sedentary behavior, ECW/ICW: estimated extracellular water to intracellular water ratio, Re/Ri (0-∞): extracellular to intracellular water resistance ratio calculated at the estimated frequency of 0 and ∞, Ze/Zi (5–250): extracellular to intracellular water resistance ratio calculated at the frequency of 5 and 250, Fc: characteristic frequency, Cm: membrane capacitance, BCM: body cell mass, FFM: fat-free mass.

**Fig. 2:**
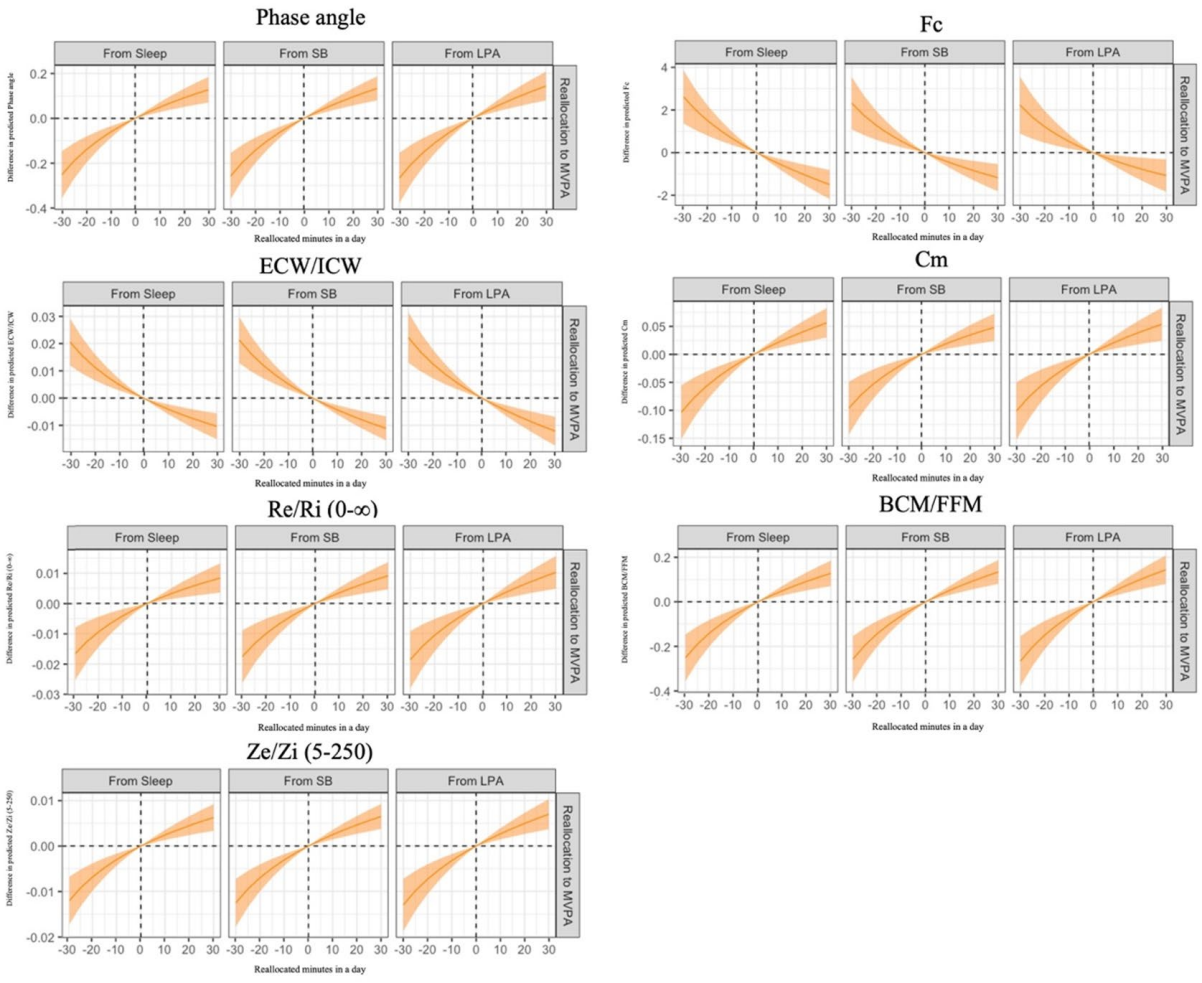
Predicted differences in electrical property-derived muscle quality when fixed amounts of time were reallocated between moderate-to-vigorous-intensity physical activity and another behavior. Solid lines represent point estimates, and shaded areas indicate 95% confidence intervals. The analysis based on the regression model included the following variables as covariates: age, sex, body mass index, smoking status, history of cancer, renal insufficiency, high blood pressure, dyslipidemia, and diabetes. MVPA: moderate-to-vigorous-intensity physical activity, LPA: light-intensity physical activity, SB: sedentary behavior, ECW/ICW: estimated extracellular water to intracellular water ratio, Re/Ri (0-∞): extracellular to intracellular water resistance ratio calculated at the estimated frequency of 0 and ∞, Ze/Zi (5–250): extracellular to intracellular water resistance ratio calculated at the frequency of 5 and 250, Fc: characteristic frequency, Cm: membrane capacitance, BCM: body cell mass, FFM: fat-free mass.

Table 2 presents the associations between 24-h movement behavior and electrical property-derived muscle quality indicators, including Re/Ri(0-∞), Ze/Zi(5-250), ECW/ICW, fc, Cm, BCM/FFM, and PhA. Daily time-use composition was significantly associated with electrical property-derived muscle quality. As outlined in the Methods section, the beta coefficients derived from compositional multiple linear regression indicated only the direction and statistical significance of associations, without reflecting direct effect sizes. To facilitate interpretation, compositional isotemporal substitution analyses were conducted and are illustrated in Figs. 1 and 2. Specifically, spending more time in MVPA relative to other behaviors was significantly associated with better electrical property-derived muscle quality, whereas time spent in LPA, SB, and sleep demonstrated no significant associations.

**Table 2:** Associations between 24-h movement behaviors and electrical property-derived muscle quality.

PhA		Sum Sq	df	F value	p-value	Model R^2^	p-value
	**Overall**	**7.295**	**3**	**8.295**	**< 0.001**	0.535	< 0.001
		Beta	95%CI		p-value		
	Sleep	0.01	(−0.351, 0.37)		0.958		
	**MVPA**	**0.315**	**(0.187**,** 0.443)**		**< 0.001**		
	LPA	−0.2	(−0.408, 0.159)		0.133		
	SB	−0.125	(−0.46, 0.061)		0.388		
ECW/ICW		Sum Sq	df	F value	p-value	Model R^2^	p-value
	**Overall**	**0.049**	**3**	**8.241**	**< 0.001**	0.469	< 0.001
		Beta	95%CI		p-value		
	Sleep	−0.004	(−0.034, 0.026)		0.779		
	**MVPA**	**−0.026**	**(−0.037**,** −0.015)**		**< 0.001**		
	LPA	0.019	(−0.012, 0.035)		0.081		
	SB	0.011	(−0.002, 0.041)		0.351		
Re/Ri (0-∞)		Sum Sq	df	F value	p-value	Model R^2^	p-value
	**Overall**	**0.033**	**3**	**5.228**	**0.002**	0.457	< 0.001
		Beta	95%CI		p-value		
	Sleep	0.006	(−0.024, 0.037)		0.675		
	**MVPA**	**0.021**	**(0.011**,** 0.032)**		**< 0.001**		
	LPA	−0.019	(−0.032, 0.015)		0.083		
	SB	−0.008	(−0.041, 0.003)		0.488		
Ze/Zi (5–250)		Sum Sq	df	F value	p-value	Model R^2^	p-value
	**Overall**	**0.017**	**3**	**7.456**	**< 0.001**	0.541	< 0.001
		Beta	95%CI		p-value		
	Sleep	0.000	(−0.019, 0.018)		0.973		
	**MVPA**	**0.015**	**(0.009**,** 0.022)**		**< 0.001**		
	LPA	−0.009	(−0.02, 0.009)		0.166		
	SB	−0.006	(−0.023, 0.004)		0.452		
Fc		Sum Sq	df	F value	p-value	Model R^2^	p-value
	**Overall**	**974.8**	**3**	**7.796**	**< 0.001**	0.437	< 0.001
		Beta	95%CI		p-value		
	Sleep	4.776	(0.479, 9.073)		0.029		
	**MVPA**	**−2.906**	**(−4.431**,** −1.381)**		**< 0.001**		
	LPA	−1.489	(−3.764, 3.002)		0.347		
	SB	−0.381	(−4.6, 1.621)		0.825		
Cm		Sum Sq	df	F value	p-value	Model R^2^	p-value
	**Overall**	**1.163**	**3**	**6.26**	**< 0.001**	0.719	< 0.001
		Beta	95%CI		p-value		
	Sleep	−0.106	(−0.271, 0.06)		0.211		
	**MVPA**	**0.123**	**(0.064**,** 0.182)**		**< 0.001**		
	LPA	−0.051	(−0.097, 0.164)		0.403		
	SB	0.034	(−0.171, 0.069)		0.613		
BCM/FFM		Sum Sq	df	F value	p-value	Model R^2^	p-value
	**Overall**	**7.295**	**3**	**8.295**	**< 0.001**	0.535	< 0.001
		Beta	95%CI		p-value		
	Sleep	0.01	(−0.006, 0.009)		0.958		
	**MVPA**	**0.315**	**(0.004**,** 0.009)**		**< 0.001**		
	LPA	−0.2	(−0.009, 0.003)		0.133		
	SB	−0.125	(−0.011, 0.001)		0.388		

Note. Boldface indicates statistical significance (*p* < 0.05). Beta cannot be interpreted directly or alone. MVPA: moderate-to-vigorous-intensity physical activity, LPA: light-intensity physical activity, SB: sedentary behavior, ECW/ICW: estimated extracellular water to intracellular water ratio, Re/Ri (0-∞): extracellular to intracellular water resistance ratio calculated at the estimated frequency of 0 and ∞, Ze/Zi (5–250): extracellular to intracellular water resistance ratio calculated at the frequency of 5 and 250, Fc: characteristic frequency, Cm: membrane capacitance, BCM: body cell mass, FFM: fat-free mass. The time-use composition is expressed as isometric log ratio (ilr) coordinates, and each result is obtained from the initial ilr coordinates. The model was adjusted for age, sex, body mass index, smoking status, history of cancer, high blood pressure, dyslipidemia, and diabetes.

The results of the MVPA-to-remaining reallocation analysis (Fig. 1) indicated that reallocating time to MVPA at the expense of all other behaviors was associated with better electrical property-derived muscle quality. The complete findings of the one-to-remaining reallocations were summarized in Supplementary Fig. 1. Specific values for time reallocations (i.e., 10, 20, and 30 min/day) from other behaviors to MVPA and vice versa were presented in Supplementary Table 1.

Figure 2 illustrates the results of MVPA-to-other reallocations, showing the effects of increasing MVPA while decreasing another behavior (e.g., SB) and vice versa. Regardless of whether LPA, SB, or sleep was replaced by MVPA, electric property-derived muscle quality was predicted to be higher. The full results of the one-to-another reallocation analysis were summarized in Supplementary Fig. 2. Specific values for time reallocations (i.e., 10, 20, and 30 min/day) between MVPA and LPA, SB, or sleep, as well as other one-to-one reallocations (e.g., from LPA to SB), were provided in Supplementary Table 2.

### Association between 24-h movement behaviors and electrical property-derived muscle quality and the effect of time reallocation in the non-regular exercise group

Table 3 and Supplementary Figs. 1 and 2 show the results of the same analysis conducted for the non-regular exercise group. Similar trends were observed in this group as in the analysis of all participants. Specifically, greater time spent in MVPA was associated with better electrical property-derived muscle quality, and reallocating time to MVPA resulted in better muscle quality derived from these properties. The results of MVPA-to-remaining and MVPA-to-other behavior reallocations are presented in Supplementary Figs. 3 and 4. Comprehensive results of one-to-remaining and one-to-other reallocations are summarized in Supplementary Figs. 5 and 6. Specific values for time reallocations (i.e., 10, 20, or 30 min/day) from remaining behaviors to MVPA, as well as from MVPA to remaining behaviors, are presented in Supplementary Table 3.

**Table 3:** Associations between 24-h movement behaviors and electrical property-derived muscle quality in the non-regular exercise group.

PhA		Sum Sq	df	F value	p-value	Model R2	p-value
	**Overall**	**4.831**	**3**	**6.137**	**0.001**	0.632	< 0.001
		Beta	95%CI		p-value		
	Sleep	0.038	(−0.573, 0.648)		0.903		
	**MVPA**	**0.396**	**(0.171**,** 0.621)**		**0.001**		
	LPA	−0.04	(−0.872, 0.086)		0.861		
	SB	−0.393	(−0.496, 0.416)		0.106		
ECW/ICW		Sum Sq	df	F value	p-value	Model R2	p-value
	**Overall**	**0.044**	**3**	**7.605**	**< 0.001**	0.602	< 0.001
		Beta	95%CI		p-value		
	Sleep	−0.005	(−0.058, 0.047)		0.838		
	**MVPA**	**−0.039**	**(−0.058**,** −0.019)**		**< 0.001**		
	LPA	0.007	(−0.004, 0.078)		0.726		
	SB	0.037	(−0.032, 0.046)		0.076		
Re/Ri(0-∞)		Sum Sq	df	F value	p-value	Model R2	p-value
	**Overall**	**0.024**	**3**	**5.019**	**0.003**	0.598	< 0.001
		Beta	95%CI		p-value		
	Sleep	0.01	(−0.038, 0.058)		0.683		
	**MVPA**	**0.029**	**(0.011**,** 0.046)**		**0.002**		
	LPA	−0.006	(−0.07, 0.005)		0.731		
	SB	−0.032	(−0.042, 0.03)		0.093		
Ze/Zi(5–250)		Sum Sq	df	F value	p-value	Model R2	p-value
	**Overall**	**0.011**	**3**	**5.614**	**0.001**	0.637	< 0.001
		Beta	95%CI		p-value		
	Sleep	0.00	(−0.031, 0.031)		0.979		
	**MVPA**	**0.019**	**(0.008**,** 0.03)**		**0.001**		
	LPA	−0.001	(−0.042, 0.007)		0.933		
	SB	−0.018	(−0.024, 0.022)		0.152		
Fc		Sum Sq	df	F value	p-value	Model R2	p-value
	**Overall**	**553.94**	**3**	**3.714**	**0.014**	0.468	< 0.001
		Beta	95%CI		p-value		
	**Sleep**	**8.406**	**(0.007**,** 16.805)**		**0.050**		
	MVPA	−2.199	(−5.291, 0.894)		0.161		
	LPA	−5.078	(−7.72, 5.461)		0.111		
	SB	−1.13	(−11.353, 1.196)		0.734		
Cm		Sum Sq	df	F value	p-value	Model R2	p-value
	**Overall**	**0.565**	**3**	**3.766**	**0.013**	0.774	< 0.001
		Beta	95%CI		p-value		
	Sleep	−0.193	(−0.46, 0.073)		0.153		
	**MVPA**	**0.128**	**(0.03**,** 0.226)**		**0.011**		
	LPA	0.005	(−0.148, 0.27)		0.961		
	SB	0.061	(−0.194, 0.204)		0.565		
BCM/FFM		Sum Sq	df	F value	p-value	Model R2	p-value
	**Overall**	**0.003**	**3**	**6.607**	**< 0.001**	0.592	< 0.001
		Beta	95%CI		p-value		
	Sleep	0.002	(−0.011, 0.016)		0.753		
	**MVPA**	**0.009**	**(0.004**,** 0.014)**		**< 0.001**		
	LPA	−0.002	(−0.02, 0.001)		0.726		
	SB	−0.01	(−0.012, 0.008)		0.075		

Note. Boldface indicates statistical significance (*p* < 0.05). Beta cannot be interpreted directly or alone. SD: standard deviation, BMI: body mass index, MVPA: moderate- to-vigorous-intensity physical activity, LPA: light-intensity physical activity, SB: sedentary behavior, ECW/ICW: estimated extracellular water to intracellular water ratio, Re/Ri (0-∞): extracellular to intracellular water resistance ratio calculated at the estimated frequency of 0 and ∞, Ze/Zi (5–250): extracellular to intracellular water resistance ratio calculated at the frequency of 5 and 250, Fc: characteristic frequency, Cm: membrane capacitance, BCM: body cell mass, FFM: fat-free mass. The time-use composition is expressed as isometric log ratio (ilr) coordinates, and each result is obtained from the initial ilr coordinates. The model was adjusted for age, sex, body mass index, smoking status, history of cancer, high blood pressure, dyslipidemia, and diabetes.

Additionally, specific reallocations values (i.e., 10, 20, or 30 min/day) from LPA, SB, or sleep to MVPA, and vice versa, as well as other one-to-one reallocations (e.g., from LPA to SB), are presented in Supplementary Table 4.

## Discussion

### Principal findings

This investigation was the first to assess the association between SB, LPA, MVPA, sleep, and muscle quality derived from electrical properties measured via BIS, including Re/Ri(0–∞), Ze/Zi(5–250), ECW/ICW, characteristic frequency, Cm, BCM/FFM, and PhA. The association was analyzed using CoDA, which appropriately accounts for the co-dependent nature of 24-h movement behaviors. The findings revealed three key points. First, the composition of 24-h movement behaviors was associated with overall muscle quality derived from electrical properties. Second, spending more time in MVPA was associated with better muscle quality. Third, reallocating hypothetical time from SB, LPA, and/or sleep to MVPA was associated with better muscle quality, whereas time spent in LPA, SB, and sleep alone was not significantly associated with muscle quality. These findings were consistent with observations in the sedentary group, which did not engage in regular PA. Collectively, the results suggest that incorporating higher levels of MVPA into daily routines may enhance muscle quality, even among individuals who do not engage in regular PA.

### Comparison with previous studies

Previous studies have demonstrated that resistance, aerobic, and combined training improve muscular and physical parameters^36^. A 24-week resistance training intervention demonstrated improvements in muscle quality, as assessed by BIS-derived electrical properties^11^. However, these exercise modalities often require substantial time, physical space, and external supervision. In contrast, MVPA integrated into daily life activities (e.g., walking at a slightly faster pace and using stairs) is more feasible and accessible. MVPA, step count, and general physical activity have been positively associated with muscle quality indicators such as intramuscular fat infiltration, PhA, and body composition^17,18,37,38^. Although the positive association between MVPA and muscle quality has been previously reported, the present study expands on this evidence by incorporating BIS-derived markers—including PhA, Cm, fc, ECW/ICW, and BCM/FFM—with CoDA. The application of CoDA enabled analysis that accounted for the compositional nature of daily time-use components and elucidated how the distribution of movement behaviors is associated with underlying muscle quality. Notably, these associations were also identified in individuals lacking structured exercise habits, further supporting the role of lifestyle-integrated MVPA in preserving muscle cell integrity.

The clinical relevance of our findings should also be considered. A recent systematic review that examined the effects of resistance training on PhA reported improvements of approximately 0.1—0.5 degrees, depending on the type and duration of the intervention^12^. In a previous intervention study, Otsuka et al. implemented a 24-week resistance training program, conducted three times per week, involving middle-aged and older adults. The study compared the effects of low- and moderate-intensity resistance training (Low-Ex and Moderate-Ex). The reported improvements in BIS-derived indices are as follows: Re/Ri(0–∞): Low-Ex: Men: 4.1% ± 4.0; Women: 2.0% ± 8.3; Moderate-Ex: Men: 5.0% ± 7.2; Women: 5.5% ± 7.0 Cm: Low-Ex: Men: 0.1% ± 7.6; Women: 2.5% ± 10.2; Moderate-Ex: Men: 8.5% ± 9.8; Women: 4.2% ± 12.5. PhA: Low-Ex: men: 2.6% ± 3.7; women: 1.6% ± 6.7; Moderate-Ex: men: 4.4% ± 4.7; women: 4.6% ± 5.9. When these values were compared with the current findings, the estimated increases in muscle quality resulted from reallocating 30 min to MVPA from other behaviors are generally fell between the effects observed in the Low-Ex and Moderate-Ex groups. In contrast, the estimated decreases in muscle quality associated with reallocating 30 min away from MVPA were comparable in Moderate-Ex group. Although the present study did not assess longitudinal changes, the observed association between daily MVPA and muscle quality suggests that even habitual, unstructured physical activity may contribute to cellular health. This finding is particularly relevant given the feasibility and accessibility of MVPA relative to structured exercise programs.

Notably, studies reporting specific quantitative changes in BIS values and corresponding health risk modifications among healthy older adults are limited. Findings from our follow-up study^39^ suggested a clinically relevant association. Specifically, a 2.5% increase in PhA from the median value of 5.08°, reflecting theoretical substitution of other behaviors with MVPA, was associated with an HR of 0.94 for functional disability. Conversely, a 4.9% decrease in PhA, reflecting substitution of MVPA with other behaviors, suggested an elevated risk (HR 1.12); however, this correlation was not statistically significant. Previously, dose-response relationships among PhA and ECW/ICW and the incidence of mortality, functional disability, and cardiovascular disease (CVD) have been reported^40^. These studies indicated that declines in these parameters increase health risks, whereas consistent risk reduction following an improvement in PhA or ECW/ICW has not been firmly established. Collectively, these findings highlighted the importance of maintaining BIS-derived muscle quality parameters to minimize health risks. Our findings showed that MVPA plays a key role in preserving muscle quality as reflected by BIS parameters. However, given the scarcity of intervention studies linking these variables to long-term outcomes, further studies are warranted to validate these associations.

### Potential mechanisms

The observed association between MVPA and improved muscle quality, particularly PhA, water distribution and Cm, may be partially explained by mechanisms similar to those proposed for resistance training which is a pathway due to increased muscle mass^13^. Furthermore, MVPA promotes mitochondrial biogenesis and improves mitochondrial function, leading to increased ATP production and improved regulation of oxidative balance. These effects help reduce cellular oxidative stress and inflammation, both of which are known to impair muscle cell integrity during aging^41^. Consequently, MVPA may support the stability of the phospholipid bilayer and preserve membrane functionality, thereby contributing to better cell membrane integrity and water distribution, both of which reflect cellular health. In contrast, such effects are unlikely to be elicited by LPA, suggesting that a specific intensity threshold may be necessary to induce physiological adaptations that enhance the electrical properties of muscle tissue. These findings underscore the importance of adequate physical intensity in maintaining muscle cell functionality and optimal fluid distribution.

The resistance ratio of ICW to ECW was examined using two frequency pairs (5 kHz and 250 kHz, zero and infinity), along with the ratios of estimated ICW to ECW, Cm, fc, and PhA as indicators of electrical properties. Although BIS more closely adheres more strictly to the theory, measuring reactance, especially at very low or high frequencies is challenging, and R0 and R ∞ calculated by the extrapolation method of curve regression may have a large margin of error. Therefore, it is reasonable to directly use the impedance values at 250 kHz and 5 kHz, which are associated with fewer sources of error^27^. Accordingly, this study employed the ICW/ECW resistance ratio using two frequency pairs (5 and 250 kHz; zero and infinity).

Muscle cell membranes, composed of phospholipid bilayers, function as capacitors that isolate the intracellular compartment from electrical currents at low frequencies. At higher frequencies, these membranes become permeable to current, enabling both ICW and ECW to contribute to electrical conductance. The application of a range of frequencies to appendicular muscle groups allow for distinct assessment of resistance in both intracellular and extracellular conductivity pathways^42,43^. Several studies have investigated the association between the estimated ECW/ICW ratio and the resistance ratio derived from ECW/ICW calculations using BIS and bioelectrical impedance analysis, in relation to muscle strength, aerobic capacity, and physical function^7–9,30,44^. Additional variables have recently been examined within the field of geriatric gerontology. Cm reflects both the holding capacity of the membrane potential gradient and the depolarization reactivity of muscle cell membranes. Fc is determined by the energy required to pass a constant current through tissue and reflects tissue density heterogeneity. PhA is defined by the phase difference (time delay) between voltage and current at the cellular and tissue levels and reflects cell mass, structural integrity, and membrane composition. Both Cm and PhA decrease with age, whereas Fc increases with age. Furthermore, Cm and PhA show a positive association with maximal power, whereas Fc exhibits a negative association. These findings indicate substantial differences in muscle composition^43^.

Muscle function declines more rapidly with age than muscle volume does. This difference is primarily attributed to a decline in muscle quality. Muscle quality declines with age, as evidenced by muscle cell mass atrophy, increased intercellular space, and unfavorable shifts in the ECW/ICW^5^. Therefore, maintaining muscle quality for preserving physical function and reducing the risk of long-term care dependence. As the findings indicate an association between MVPA and muscle quality based on electrical properties, MVPA may exert beneficial effects on cell membrane integrity and ICW distribution. These findings offer detailed insights into the cellular mechanisms underlying the beneficial effects of MVPA. However, as muscle quality derived from electrical properties is grounded in electrical theory and subject to potential measurement, calculation, and estimation errors, validation through anatomical methods is necessary.

Our results provide important evidence that MVPA is positively associated with better muscle quality as determined through electrical properties. This association was also evident among individuals in the non-exercise group. Although resistance training remains effective, its practical implementation is often challenging. In contrast, daily MVPA is feasible to implement, though its effect on body composition among older adults remain unclear. Therefore, our findings indicate that higher levels of daily MVPA are associated with high muscle quality, offering valuable evidence supporting MVPA as a modifiable behavioral intervention.

### Limitations

However, investigations are warranted due to several methodological limitations. First, as this study employed a cross-sectional design, it was not possible to determine whether there is a causal relationship between exercise behavior and muscle quality as determined by electrical properties. Higher levels of MVPA may be a result of better muscle quality, suggesting the possibility of reverse causation. Furthermore, both variables may be influenced by unmeasured confounding factors, such as dietary patterns, sleep quality, comorbidities, medication use, socioeconomic status, or genetic predisposition. Longitudinal and interventional studies are required to confirm causality. Second, the potential for measurement error must be considered. Although the methodology was adapted from previous studies^32^, sleep duration was assessed through self-reported bedtimes and wake times, potentially introducing recall bias and reporting inaccuracies. While this approach is widely used in CoDA studies, the potential for misclassification persists and may contribute to non-differential measurement error. Additionally, due to limitations of the accelerometer employed, activities with intensity below 1.1 METs could not be differentiated from periods of no signal. Consequently, SB was estimated by subtracting MVPA and LPA from total waking hours, in accordance with methods described in prior studies using the same device^24^. However, this approach may result in misclassification of SB and introduce non-differential measurement error, potentially leading to underestimation or overestimation of actual sedentary time. Therefore, findings related to SB should be interpreted with caution. Third, theoretical isotemporal substitution, such as the replacement of MVPA with other activity behaviors, were estimated. As these results are derived from statistical estimates, specific values, and their interpretations should be approached with caution. Fourth, the study population primarily consisted of community-dwelling older Japanese adults, with a relatively high representation of women. Such sampling bias may restrict the generalizability of the findings to other demographic groups, particularly non-Asian or more gender-balanced cohorts. Moreover, individuals who consented to participate may have exhibited greater health consciousness or physical activity than the general population, potentially influencing the observed associations. Finally, employing BIS to estimate ICW, ECW, and BCM represents a methodological limitation. BIS is a secondary, indirect method subject to interindividual variability and influenced by transient fluctuations in body hydration. The gold-standard techniques for estimating these parameters include deuterium and sodium dilution methods, along with whole-body counting. Nevertheless, BIS remains a widely utilized, noninvasive, and practical instrument for evaluating body fluid distribution in both research and clinical settings.

## Conclusion

This cross-sectional study found a positive association between MVPA and muscle quality, as determined by electrical properties measured using BIS. A longer time spent in MVPA was associated with more favorable BIS-derived muscle quality indices. Theoretical time reallocations from sleep, sedentary behavior or light-intensity physical activity to MVPA were associated with better muscle quality. These findings should be interpreted as associations rather than as indicating longitudinal changes. Similar associations were observed among individuals without regular exercise habits. These findings suggest that MVPA may be linked to BIS-derived muscle quality in daily life, although longitudinal and interventional studies are needed to clarify causality.

## Supplementary Information

Below is the link to the electronic supplementary material.

Supplementary Material 1

Supplementary Material 2

Supplementary Material 3

Supplementary Material 4

Supplementary Material 5

Supplementary Material 6

## Data Availability

The data are not publicly available because of privacy and ethical restrictions. The data supporting the findings of this study are available from the corresponding author upon request.

## Abbreviations

BCM: Body cell mass
BIS: Bioelectrical impedance spectroscopy
BMI: Body mass index
Cm: Membrane capacitance
CoDA: Compositional data analysis
ECW: Extracellular water
fc: Characteristic frequency
FFM: Fat-free mass
ICW: Intracellular water
ILR: Isometric log ratio
LPA: Light-intensity physical activity
MET: Metabolic equivalent of task
MVPA: Moderate-to-vigorous-intensity physical activity
PA: Physical activity
PhA: Phase angle
SB: Sedentary behavior
