# Supplementary material for: Relationship between bioelectrical impedance–derived muscle quality and physical activity, sedentary behavior, or sleep using compositional data analysis (CoDA)
Source: Sci Rep. 2025 Dec 18;15:44108. doi: 10.1038/s41598-025-27953-3 (PMC12714699; doi:10.1038/s41598-025-27953-3)
Supplement: Supplementary file 6 — Supplementary Material 6 [file 41598_2025_27953_MOESM6_ESM.docx]

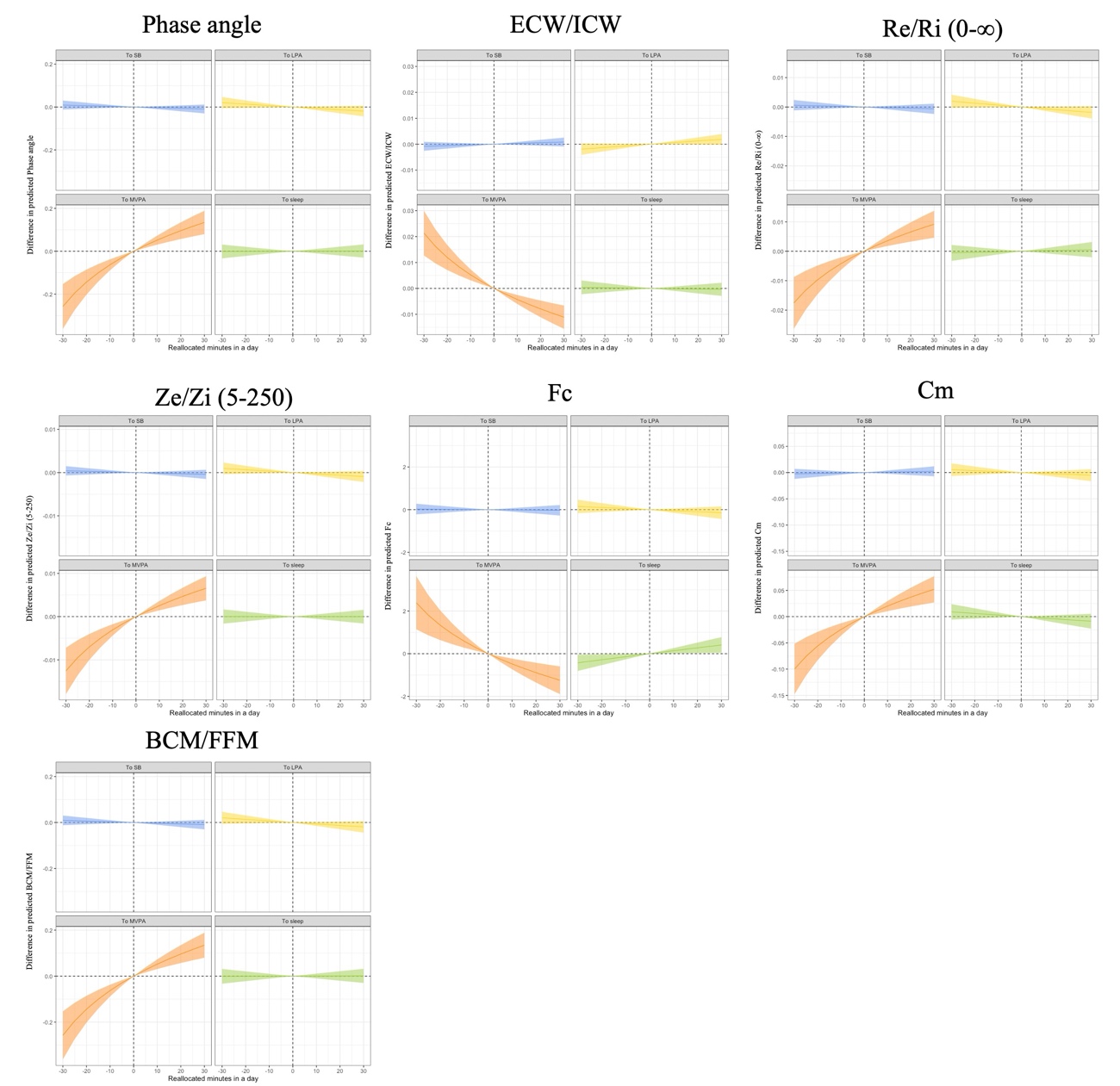


Supplementary Figure 1. Predicted differences in electric property-derived muscle quality when fixed amounts of time were reallocated between one behavior to remaining behaviors, while keeping the reaming components constant at compositional means. The analysis based on the regression model that included the following variables as covariates: age, sex, body mass index, smoking status, history of cancer, renal insufficiency, high blood pressure, dyslipidemia, and diabetes. MVPA: moderate- to vigorous-intensity physical activity, LPA: light-intensity physical activity, SB: sedentary behavior, ECW/ICW: estimated extracellular to intracellular water ratio, Re/Ri (0-∞): extracellular to intracellular water resistance ratio calculated at the estimated frequency of 0 and ∞, Ze/Zi (5-250): extracellular to intracellular water resistance ratio calculated at the frequency of 5 and 250, Fc: characteristic frequency, Cm: membrane capacitance, BCM: body cell mass, FFM: fat free mass.


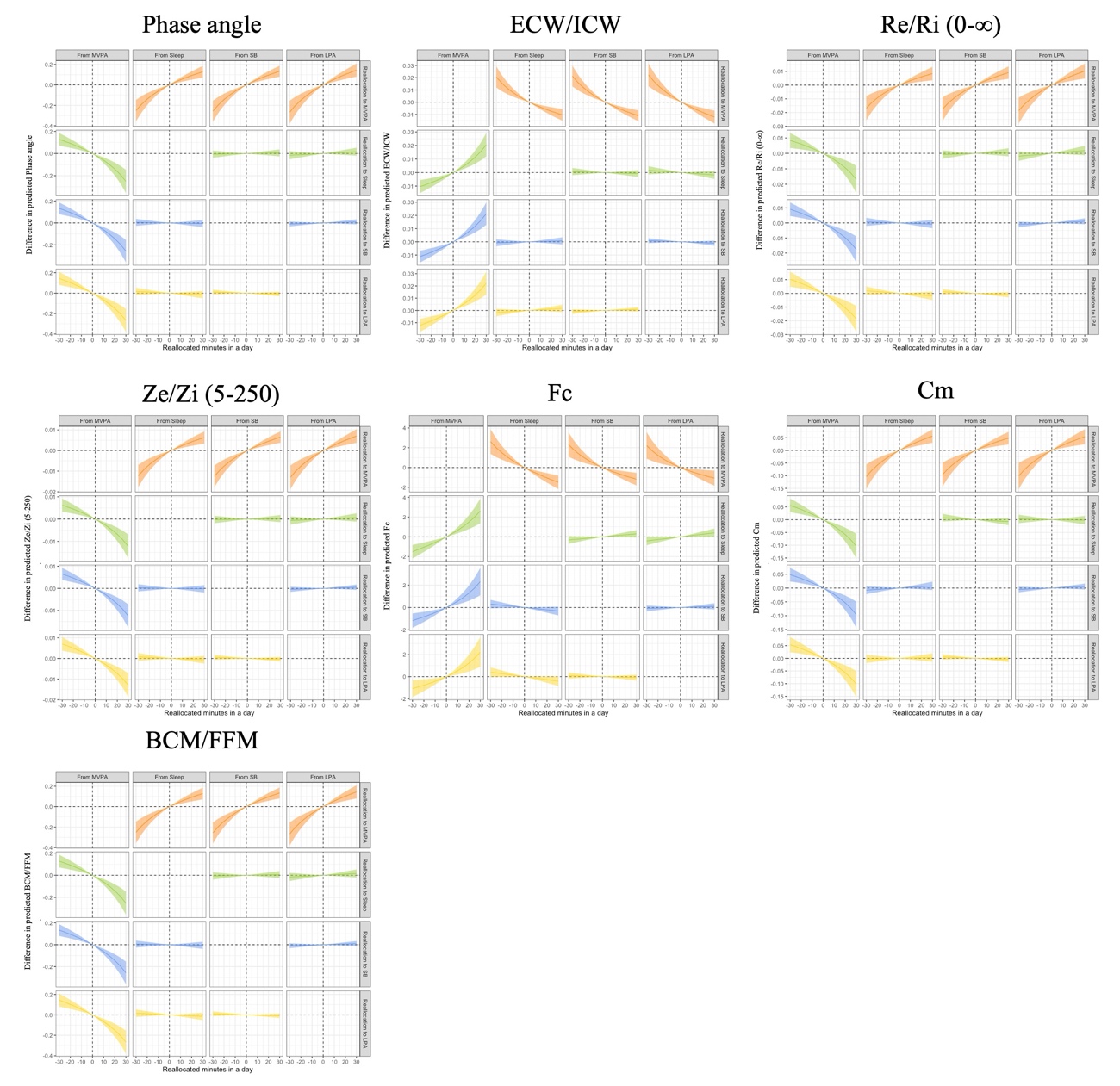


Supplementary Figure 2. Predicted differences in electric property-derived muscle quality when fixed amounts of time were reallocated between one behavior and another behavior. The analysis based on the regression model that included the following variables as covariates: age, sex, body mass index, smoking status, history of cancer, high blood pressure, dyslipidemia, and diabetes. MVPA: moderate- to vigorous-intensity physical activity, LPA: light-intensity physical activity, SB: sedentary behavior, ECW/ICW: estimated extracellular to intracellular water ratio, Re/Ri (0-∞): extracellular to intracellular water resistance ratio calculated at the estimated frequency of 0 and ∞, Ze/Zi (5-250): extracellular to intracellular water resistance ratio calculated at the frequency of 5 and 250, Fc: characteristic frequency, Cm: membrane capacitance, BCM: body cell mass, FFM: fat free mass.


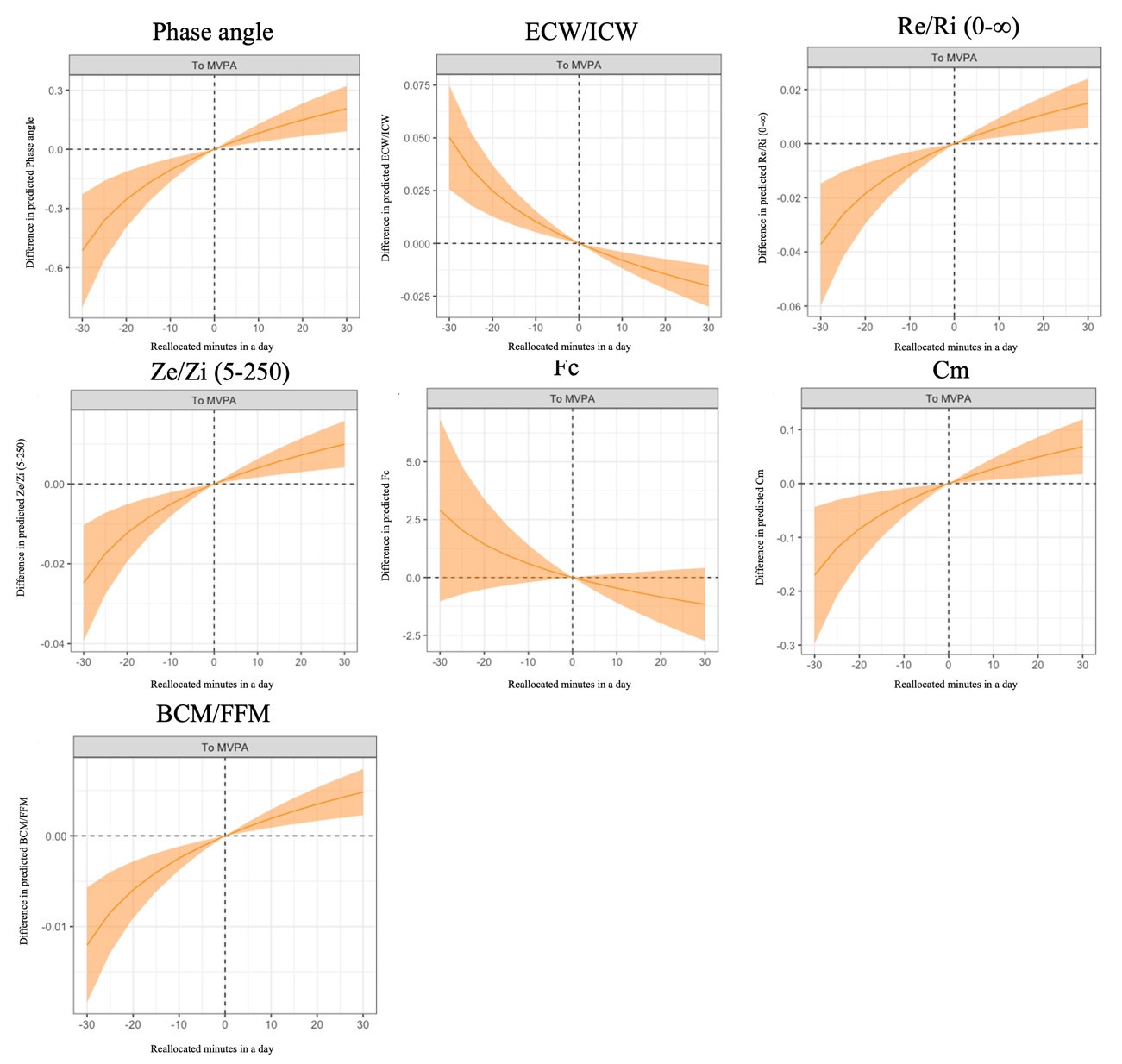


Supplementary Figure 3. In the non-regular exercise group, predicted differences in electric property-derived muscle quality when fixed amounts of time were reallocated between moderate- to vigorous-intensity physical activity and remaining behaviors, while keeping the reaming components constant at compositional means. The analysis based on the regression model that included the following variables as covariates: age, sex, body mass index, smoking status, history of cancer, high blood pressure, dyslipidemia, and diabetes. MVPA: moderate- to vigorous-intensity physical activity, LPA: light-intensity physical activity, SB: sedentary behavior, ECW/ICW: estimated extracellular to intracellular water ratio, Re/Ri (0-∞): extracellular to intracellular water resistance ratio calculated at the estimated frequency of 0 and ∞, Ze/Zi (5-250): extracellular to intracellular water resistance ratio calculated at the frequency of 5 and 250, Fc: characteristic frequency, Cm: membrane capacitance, BCM: body cell mass, FFM: fat free mass.


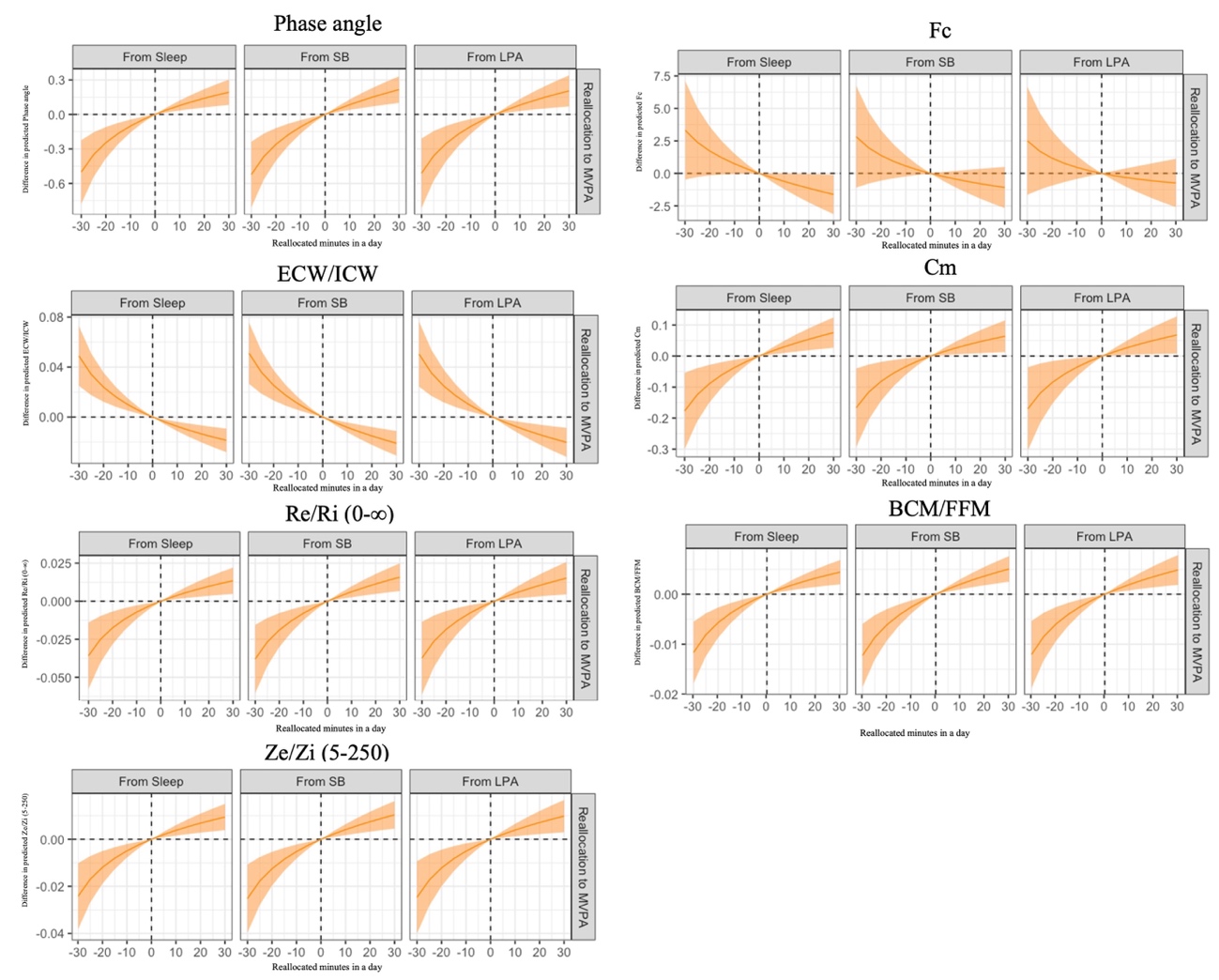


Supplementary Figure 4. In the non-regular exercise group, predicted differences in electric property-derived muscle quality when fixed amounts of time were reallocated between moderate- to vigorous-intensity physical activity and another behavior. The analysis based on the regression model including the following variables as covariates: age, sex, body mass index, smoking status, history of cancer, high blood pressure, dyslipidemia, and diabetes. MVPA: moderate- to vigorous-intensity physical activity, LPA: light-intensity physical activity, SB: sedentary behavior, ECW/ICW: estimated extracellular to intracellular water ratio, Re/Ri (0-∞): extracellular to intracellular water resistance ratio calculated at the estimated frequency of 0 and ∞, Ze/Zi (5-250): extracellular to intracellular water resistance ratio calculated at the frequency of 5 and 250, Fc: characteristic frequency, Cm: membrane capacitance, BCM: body cell mass, FFM: fat free mass.


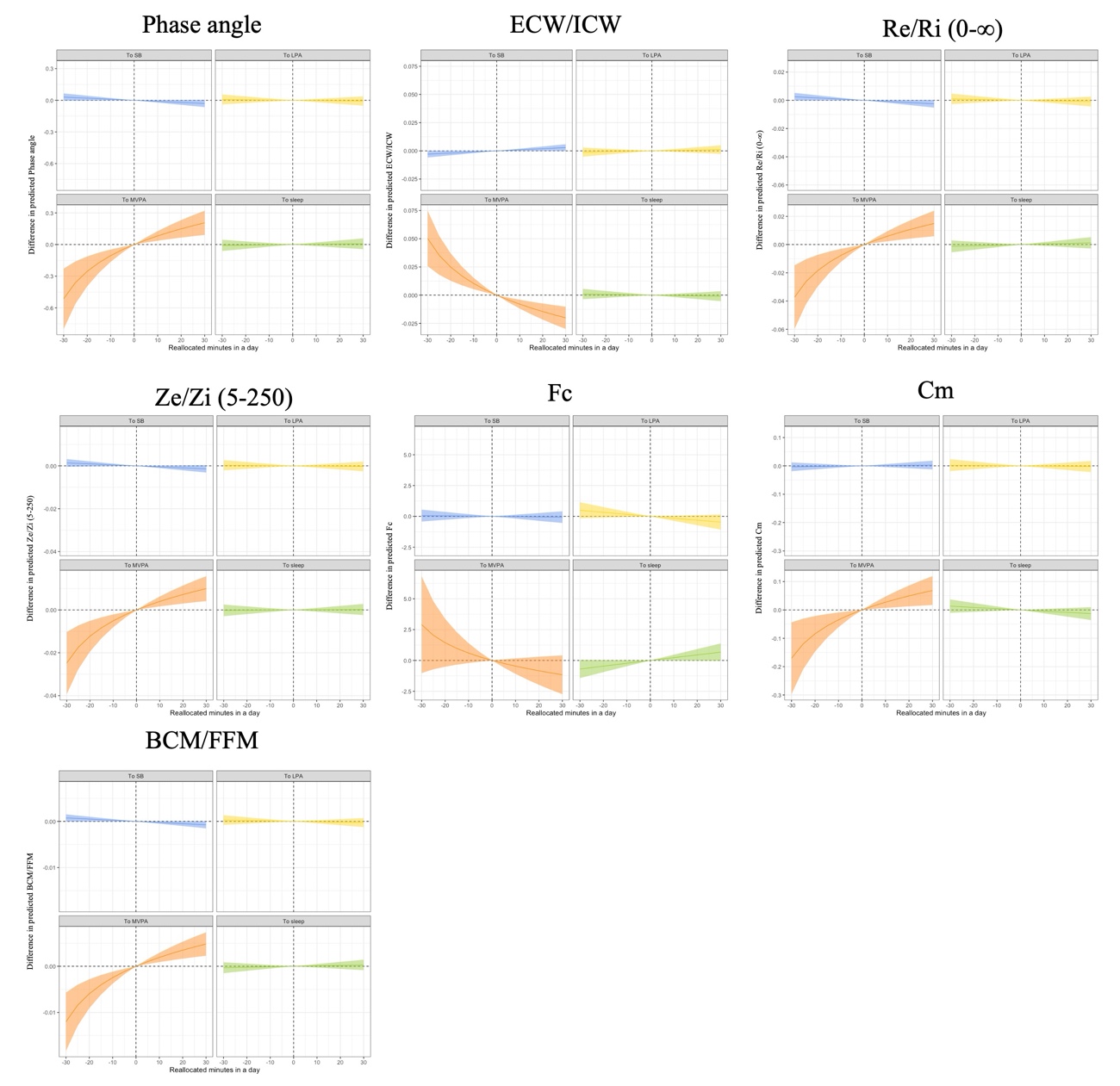


Supplementary Figure 5. In the non-regular exercise group, predicted differences in electric property-derived muscle quality when fixed amounts of time were reallocated between one behavior to remaining behaviors, while keeping the reaming components constant at compositional means. The analysis based on the regression model that included the following variables as covariates: age, sex, body mass index, smoking status, history of cancer, renal insufficiency, high blood pressure, dyslipidemia, and diabetes. MVPA: moderate- to vigorous-intensity physical activity, LPA: light-intensity physical activity, SB: sedentary behavior, ECW/ICW: estimated extracellular to intracellular water ratio, Re/Ri (0-∞): extracellular to intracellular water resistance ratio calculated at the estimated frequency of 0 and ∞, Ze/Zi (5-250): extracellular to intracellular water resistance ratio calculated at the frequency of 5 and 250, Fc: characteristic frequency, Cm: membrane capacitance, BCM: body cell mass, FFM: fat free mass.


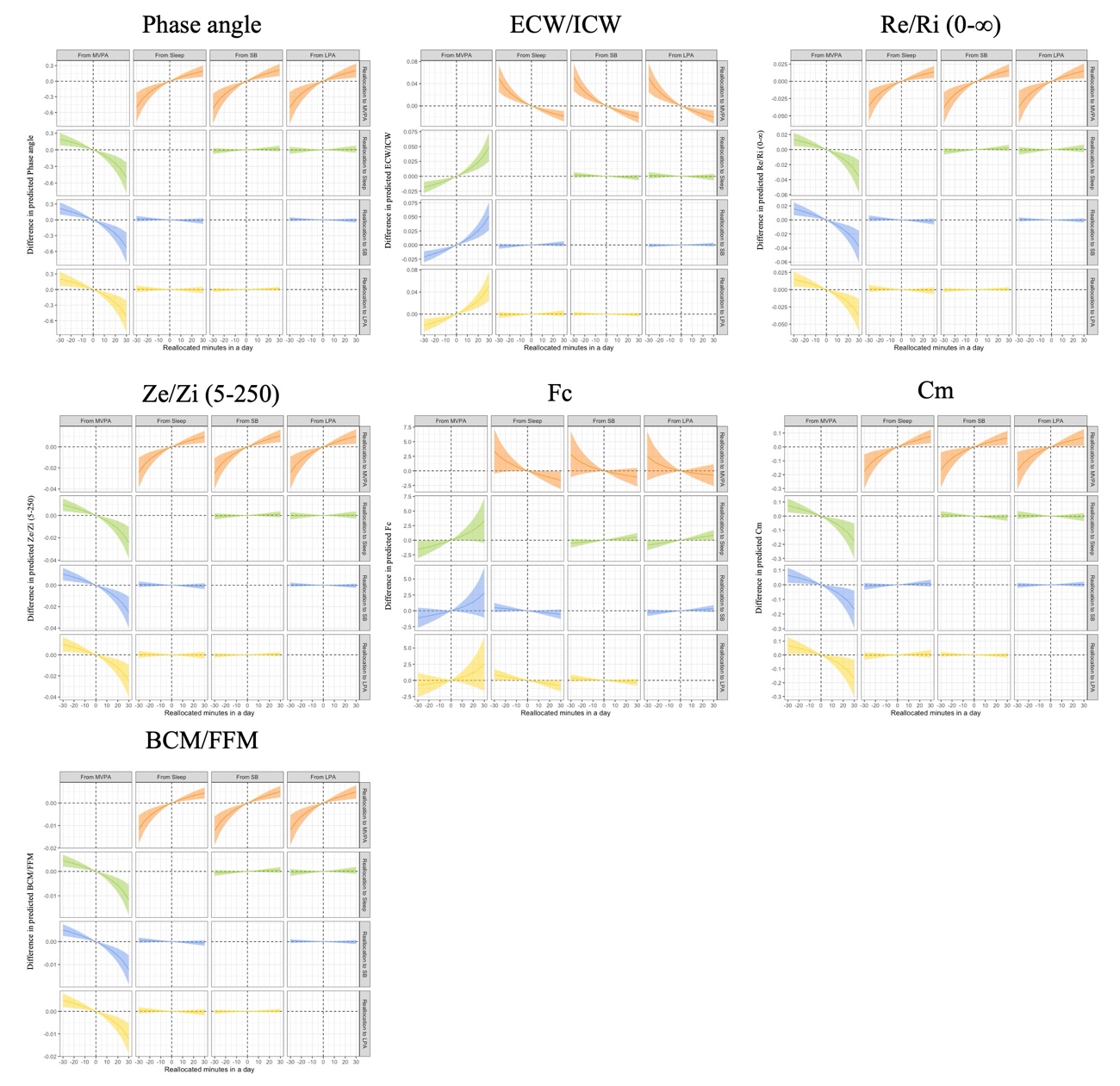


Supplementary Figure 6. In the non-regular exercise group, predicted differences in electric property-derived muscle quality when fixed amounts of time were reallocated between one behavior and another behavior. The analysis based on the regression model that included the following variables as covariates: age, sex, body mass index, smoking status, history of cancer, high blood pressure, dyslipidemia, and diabetes. MVPA: moderate- to vigorous-intensity physical activity, LPA: light-intensity physical activity, SB: sedentary behavior, ECW/ICW: estimated extracellular to intracellular water ratio, Re/Ri (0-∞): extracellular to intracellular water resistance ratio calculated at the estimated frequency of 0 and ∞, Ze/Zi (5-250): extracellular to intracellular water resistance ratio calculated at the frequency of 5 and 250, Fc: characteristic frequency, Cm: membrane capacitance, BCM: body cell mass, FFM: fat free mass.


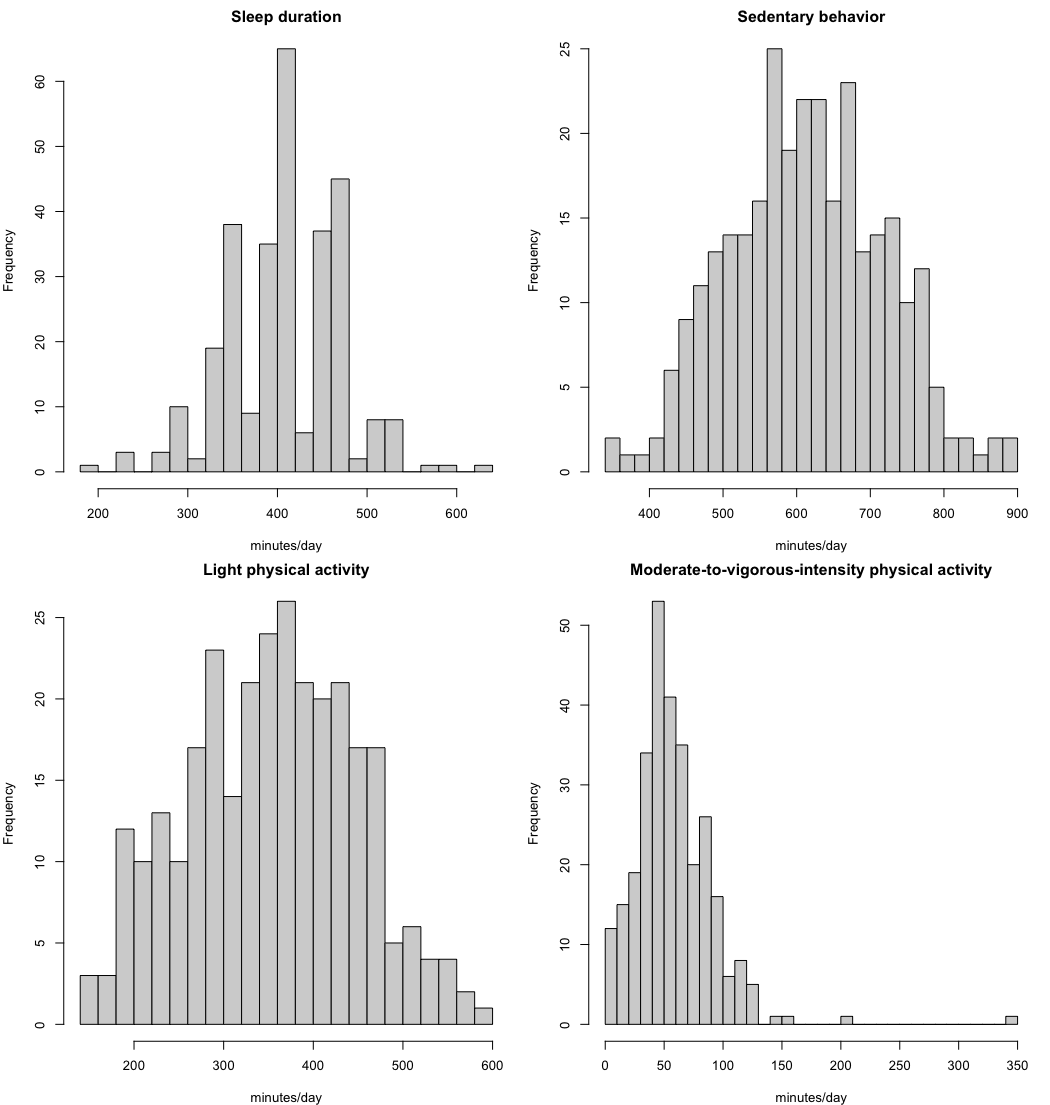


Supplementary Figure 7. The distribution of movement behaviors.
